# Manipulation of Autophagy and Apoptosis Facilitates Intracellular Survival of *Staphylococcus aureus* in Human Neutrophils

**DOI:** 10.3389/fimmu.2020.565545

**Published:** 2020-11-11

**Authors:** Michelle E. Mulcahy, Eóin C. O’Brien, Kate M. O’Keeffe, Emilio G. Vozza, Neal Leddy, Rachel M. McLoughlin

**Affiliations:** ^1^ Host-Pathogen Interactions Group, School of Biochemistry and Immunology, Trinity Biomedical Sciences Institute, Trinity College Dublin, Dublin, Ireland; ^2^ bioTEM, School of Biochemistry and Immunology, Trinity Biomedical Sciences Institute, Trinity College Dublin, Dublin, Ireland

**Keywords:** apoptosis, autophagy, neutrophils, p53, *S. aureus*

## Abstract

Polymorphonuclear neutrophils (PMN) are critical for first line innate immune defence against *Staphylococcus aureus*. Mature circulating PMN maintain a short half-life ending in constitutive apoptotic cell death. This makes them unlikely candidates as a bacterial intracellular niche. However, there is significant evidence to suggest that *S. aureus* can survive intracellularly within PMN and this contributes to persistence and dissemination during infection. The precise mechanism by which *S. aureus* parasitizes these cells remains to be established. Herein we propose a novel mechanism by which *S. aureus* subverts both autophagy and apoptosis in PMN in order to maintain an intracellular survival niche during infection. Intracellular survival of *S. aureus* within primary human PMN was associated with an accumulation of the autophagic flux markers LC3-II and p62, while inhibition of the autophagy pathway led to a significant reduction in intracellular survival of bacteria. This intracellular survival of *S. aureus* was coupled with a delay in neutrophil apoptosis as well as increased expression of several anti-apoptotic factors. Importantly, blocking autophagy in infected PMN partially restored levels of apoptosis to that of uninfected PMN, suggesting a connection between the autophagic and apoptotic pathways during intracellular survival. These results provide a novel mechanism for *S. aureus* intracellular survival and suggest that *S. aureus* may be subverting crosstalk between the autophagic and apoptosis pathways in order to maintain an intracellular niche within human PMN.

## Introduction

*Staphylococcus aureus* is a leading global cause of bloodstream infection and is associated with a higher mortality rate than other bacteraemia, typically 25% (1, 2). Treatment of *S. aureus* bloodstream infection is becoming increasingly difficult due to antibiotic resistance. Bacteraemia can lead to metastatic infection in a subset of patients (3) and *S. aureus* persistent bacteraemia and relapse of infection has been linked to survival within an intracellular reservoir (4). This ‘Trojan Horse’ theory has been implicated as a contributing factor in recurrent disease, with the indication that *S. aureus* may be particularly adept at surviving within polymorphonuclear neutrophils (PMN) (5).

PMN are critical in the innate immune response against *S. aureus* infection. Optimal PMN function is beneficial for the host during *S. aureus* infection; individuals with deficiencies in PMN activity such as those with chronic granulomatous disease suffer from recurrent *S. aureus* infections (6, 7). However, in murine models of *S. aureus* infection, high levels of PMN recruitment can contribute to dissemination and pathogenesis (8, 9) allowing *S. aureus* to survive intracellularly in a neutrophil-rich environment (10). *S. aureus* is considered a non-classical facultative intracellular pathogen (11, 12) and can survive within several cell types including keratinocytes, osteoblasts and leukocytes, including neutrophils (8, 13–15). Recently, we reported that during murine peritoneal infection, *S. aureus* was found predominantly within PMN disseminated from the peritoneal cavity to the bloodstream (16). These studies support the notion of intracellular survival in PMN as a possible bacterial virulence mechanism. This mechanism, however, is still unclear.

*S. aureus* survival in non-professional phagocytes has been linked to subversion of a cellular process called macroautophagy (hereafter called autophagy) (17, 18). Autophagy is a conserved eukaryotic process in which damaged organelles are recycled in order to create a supply of nutrients (19). Autophagy involves the *de novo* formation of a phagophore that eventually elongates to form a double-membraned phagosome, or autophagosome (20). Phagophore nucleation is initiated by an activation complex comprising of a class III PI3-kinase called vacuolar protein-sorting 34 (VPS34), Beclin-1, and ATG14 (21). Autophagosome formation is driven by the lipidation of the autophagy marker LC3-I to LC3-II (22). Targeted organelles are engulfed by the phagophore during autophagosome formation and subsequent fusion of the autophagosome with a lysosome degrades the autophagic cargo.

Previous studies have described divergent mechanisms for *S. aureus* intracellular survival and replication using the autophagic pathway in non-professional phagocytes. *S. aureus* was reported to survive and replicate in LC3-decorated autophagosomes in HeLa cells, followed by eventual escape into the cytoplasm (17). In murine fibroblasts, ubiquitinated *S. aureus* was trafficked to autophagosomes by selective autophagic chaperone proteins such as p62 but prevented autophagosome-lysosome fusion (18). Taken together, these data propose a role for autophagy during *S. aureus* invasion of non-professional phagocytes; however, a role of autophagy for *S. aureus* survival within primary human phagocytes after phagocytosis remains to be established.

PMN have a short half-life in humans (23). PMN turnover is controlled by constitutive apoptosis, making it a seemingly inadequate niche for bacterial intracellular survival. However, the normal course of apoptosis in human PMN can be modulated by *S. aureus* (24). During methicillin-resistant *S. aureus* (MRSA) infection, primary human PMN display an aberrant apoptosis phenotype (25). It is not known whether alterations in the apoptotic pathway in PMN after exposure to *S. aureus* are associated with intracellular survival.

This study explores the effect of *S. aureus* intracellular survival on the autophagic and apoptotic pathways in primary human PMN. We demonstrate that *S. aureus* intracellular survival depends on a functioning autophagic pathway and is associated with a delay in PMN apoptosis. Importantly, we have uncovered evidence that *S. aureus* may be manipulating both pathways in order to preserve an intracellular niche during bloodstream infection.

## Materials and Methods

### Bacterial Strains and Growth Conditions

*S. aureus* strains PS80, PS80Δagr, USA300 LAC and USA300 LACΔagrC have been described previously (16, 26, 27). *S. aureus* strains were streaked from frozen stocks onto TSA plates and grown at 37°C for 24 h. Bacterial suspensions were prepared in sterile PBS and the OD at 600 nm adjusted to the desired equivalent CFU/ml consistent with previous studies (9, 16, 28).

### Isolation of Primary Human Neutrophils

Neutrophils were isolated from the peripheral blood of healthy volunteers following informed consent and according to institutional ethical guidelines. Briefly, neutrophils were isolated by dextran sedimentation and gradient separation using Ficoll-Hypaque centrifugation (Lymphoprep, Axis-Shield). After erythrocyte lysis using ACK buffer (Gibco), PMN were resuspended in Dulbecco’s Modified Eagles Medium (Sigma) supplemented with 10% fetal bovine serum (Sigma) and 1% L-glutamine (Sigma). PMN were adjusted to a final concentration of 2x10^6^ cells per replicate. Following isolation, PMN purity was >95% and viability >99% as determined by flow cytometry.

### *Staphylococcus aureus* Intracellular Survival Assay

Bacteria were incubated with human IVIG (5 mg/ml, Kiovig) and Low-Tox Guinea Pig Complement (Cedarlane) for 20 min at 37°C in order to opsonise them for efficient uptake by PMN. Bacteria were then added to PMN at a MoI of 1:10. In some cases PMN were pre-treated with VPS34-IN1 (10 µM, Millipore), Bafilomycin A1 (100 nM, Sigma), Pifithrin-α (30μM, Merck) or a corresponding DMSO vehicle control for 30 min prior to inoculation with *S. aureus*. PMN were incubated with bacteria for 1 h with rotation at 37°C before addition of gentamicin (Sigma) at a final concentration of 200 µg/ml. PMN and bacteria were incubated for a further 1, 3, or 6 h with rotation before centrifugation. Gentamicin treatment was continued for the duration of the time-course to ensure no survival of extracellular bacteria. Media was plated onto TSA at 1, 3, and 6 h post gentamicin treatment to ensure that no bacteria survived extracellularly. PMN were lysed in 0.1% (v/v) Triton-X 100 (Sigma). Lysates were diluted in PBS and plated onto TSA for CFU enumeration. Under all conditions PMN viability began to decline 12 hours after blood was drawn from donors as has previously been reported (29–31).

Alternatively, to assess phagocytosis, cells were pre-treated and infected as above with GFP-expressing PS80 followed by gentamicin treatment for 30 min to eliminate extracellular bacteria. PMN were then fixed with Fix & Perm Medium A (Life Technologies) and analysed on BD FACSCanto II. The percentage of GFP-positive cells was used as an indicator of intracellular bacteria.

### RNA Extraction, cDNA Synthesis, and Quantitative PCR

Total RNA was extracted using the Qiagen RNA extraction kit according to the manufacturers’ instructions. RNA yield and quality were measured on a Spectrostar Nano spectrophotometer using an LVIS plate. RNA (250ng) was reverse-transcribed using a High-Capacity cDNA reverse transcription kit (Applied Biosystems) according to manufacturers’ instructions. mRNA was quantified using quantitative PCR on a CFX96 Touch Real-Time PCR Detection System (Bio-Rad) using iTaq Sybr Green Supermix (Bio-Rad) according to manufacturers’ recommendations. The following KiCqStart SYBR Green primer pairs (Sigma) were used: human *tp53* (Gene ID: 7157), *dram1* (Gene ID: 55332), *mcl1* (Gene ID:4170), *bcl2* (Gene ID: 596), *bcl2a1* (Gene ID:597), *bax* (Gene ID: 581), and *rn18s1* (Gene ID: 100008588). Expression was normalized to 18s RNA by the change-in-cycle-threshold (ΔΔCT) method.

### Protein Expression

PMN were lysed in lysis buffer (1% (v/v) Triton-X-100 (Sigma), 5% (v/v) protease inhibitor cocktail (Sigma) and 5% (v/v) phosphatase inhibitor cocktail 3 (Sigma)). Lysate protein concentration was determined using a Pierce BCA assay kit (Thermo Fisher Scientific). A total of 5μg of protein was separated on 4–20% precast TGX polyacrylamide gels (Bio-Rad) and transferred onto polyvinylidene difluoride membranes. Membranes were incubated in AdvanBlock (Cell Signalling Technologies) before probing with rabbit anti-human LC3 IgG (D3U4C, Cell Signalling Technologies), rabbit anti-SQSTM1/p62 IgG (5114, Cell Signalling Technologies), rabbit anti-human caspase-3 IgG (9662, Cell Signalling Technology), rabbit anti-human Mcl-1 IgG (D2W9E, Cell Signalling Technology), rabbit anti-human A1/Bfl-1 IgG (D1A1C, Cell Signalling Technology), rabbit anti-human Bax IgG (D2E11, Cell Signalling Technology), rabbit anti-human p53 IgG (7F5, Cell Signalling Technology), rabbit anti-human phospho-p53 (Ser15) IgG (9284, Cell Signalling Technology), and rabbit anti-DRAM1 (ARP47432_P050, Aviva Systems Biology). Incubation with primary antibodies was followed by HRP-conjugated goat anti-rabbit IgG (7074, Cell Signalling Technology). Reactive bands were visualized using ECL detection and densitometry was performed using ImageLab developing system (Bio-Rad).

### Flow Cytometry

To assess apoptosis, cells were stained using the Terminal deoxynucleotidyl transferase (TdT)-mediated dUTP nick end labelling (TUNEL) kit (Roche). PMN were fixed with Fix & Perm Medium A (Life Technologies) and permeabilized with nuclear permeabilization buffer before incubation with TUNEL reaction mixture according to manufacturer’s instructions. To assess mitochondrial membrane potential, cells were stained using 5,5’,6,6’-tetrachloro-1,1’,3,3’-tetraethylbenzimidazolcarbocyanine iodide (JC-1) (BD Biosciences). Membrane depolarisation is characterized by a fluorescence emission shift from green (~529 nm, FL2) to red (~590, FL1). PMN (1 X 10^6^ cells) were incubated with JC-1 for 15 min at 37°C and 5% CO_2_ according to manufacturer’s instructions. To determine intracellular p53 levels, PMN were fixed with Fix & Perm Medium A (Life Technologies) and permeabilized with nuclear permeabilization buffer before incubation with anti-p53 APC (MiltenyiBiotec) for 15 min at room temperature. All flow cytometric analysis was performed immediately with a BD FACSCanto II using FACS DIVA and FlowJo software.

### Transmission Electron Microscopy

Infected PMN samples were fixed in glutaraldehyde (1.5%) overnight at 4°C. Samples were washed and then embedded in agarose (2%). Samples were then secondary fixed with osmium tetroxide (2% in 0.05M potassium phosphate buffer) before dehydration through increasing concentrations of ethanol. Samples were transitioned with propylene oxide into epoxy resin embedding medium then cured at 60°C for 24 h. Ultrathin sections were obtained using a Leica EM UC7 ultramicrotome and transferred on to 300 mesh copper TEM grids. Sample grids were stained with 0.5% aqueous uranyl acetate and Reynold’s lead citrate. Sections were examined on a Jeol JEM1400 transmission electron microscope at 100 kV and imaged with AMT XR80 digital camera.

### Statistical Analyses

Statistical analysis was performed using Prism Graphpad 8 software using ANOVA or repeated measures ANOVA. Comparisons between groups were made using Bonferroni post-tests or Tukey post-tests where appropriate.

## Results

### *Staphylococcus aureus* Intracellular Survival Within Primary Human Neutrophils Requires the Agr Virulence Regulator and the Autophagy Pathway

**Figure 1:**
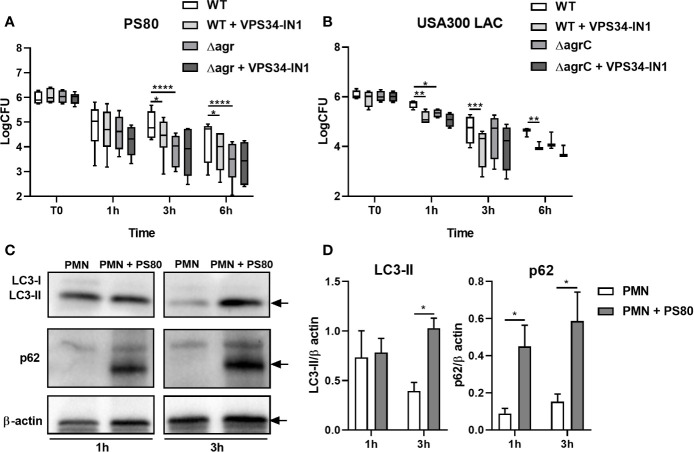
*Staphylococcus aureus* intracellular survival within human neutrophils. Primary human neutrophils were treated with VPS34-IN1 (10 µM) or were left untreated and were then infected with pre-opsonized *S. aureus* PS80 WT or Δagr **(A)** or USA300 LAC WT or ΔagrC **(B)** (MoI 10) for 1 h. Following infection, PMN were treated with gentamicin (200 µg/ml) for the times indicated. At each timepoint, PMN lysates were plated onto TSA and CFU enumerated. Data are expressed as Log CFU (n = 6 donors for A, n = 3–4 donors for B). Statistical analyses were performed using two-way ANOVA with Bonferroni post-tests. *P < 0.05; **P < 0.01; ***P < 0.001; ****P < 0.001.PMN protein lysates were probed for LC3 processing and p62 expression using Western immunoblotting **(C)** and analysed using densitometric analysis **(D)**. Data are expressed as protein expression normalized to β-actin control values for each sample ± SEM (n = 3 donors). Black arrows indicate the area of the blot used for densitometry. Statistical analyses were performed using two-way ANOVA with Bonferroni post-tests. *P < 0.05.

In order to determine the ability of *S. aureus* to survive intracellularly within primary human PMN, PMN isolated from the peripheral blood of healthy volunteers were incubated with *S. aureus* strain PS80 for 1 h before gentamicin treatment to kill any non-phagocytosed extracellular bacteria. Intracellular CFU were assessed at 1, 3, and 6 h post-gentamicin treatment. Survival of WT PS80 within PMN was significantly higher than that of PS80Δagr at 3 and 6 h post-gentamicin treatment (**Figure 1A**) highlighting the requirement for an agr-specific factor for intracellular survival.

To evaluate the role of the autophagy pathway during intracellular survival, PMN were treated with the VPS34-PI3K inhibitor VPS34-IN1 before exposure to PS80. Intracellular survival of PS80 in inhibitor-treated PMN was significantly reduced at 3 and 6 h post-gentamicin treatment compared to untreated, infected PMN (**Figure 1A**). The rate of phagocytosis of *S. aureus* by PMN was not affected by VPS34-IN1 treatment (**Figure S1**). These results indicate that inhibition of the autophagy pathway impairs the ability of *S. aureus* to survive intracellularly within human PMN. There was no further decrease in survival in PS80Δagr-infected, VPS34-IN1-treated PMN (**Figure 1A**), suggesting that an agr-specific factor is involved in manipulation of the autophagy pathway.

The assay was repeated with the USA300 strain LAC and an agrC-deficient isogenic mutant (USA300ΔagrC). Similar to PS80, intracellular survival of WT USA300 in inhibitor-treated PMN was significantly lower at each timepoint compared to untreated, infected PMN (**Figure 1B**). Intracellular CFU levels of USA300ΔagrC differed significantly from WT USA300 CFU at 1 h. Taken together, these results indicate that a functioning autophagy pathway facilitates *S. aureus* intracellular survival in PMN and that this mechanism of intracellular survival involves an agr-specific factor.

### Autophagic Flux Is Disrupted in PMN Harboring *Staphylococcus aureus*

To assess autophagic flux in PMN harboring *S. aureus*, protein levels of autophagic markers LC3-II and p62 were determined in *S. aureus*-infected PMN compared to untreated controls at 1 and 3 h post-gentamicin treatment using Western immunoblotting. After 3 h, LC3-II levels were significantly higher in *S. aureus*-infected PMN compared to control cells (**Figures 1C, D**). These data indicate that the autophagy pathway is activated in infected PMN compared to untreated cells. Levels of p62 were also significantly increased in infected PMN after 3 h gentamicin treatment compared to untreated PMN (**Figures 1C, D**) which suggests that autophagic flux has been disrupted in *S. aureus*-infected cells and that autophagosomes may be accumulating during intracellular survival.

**Figure 2:**
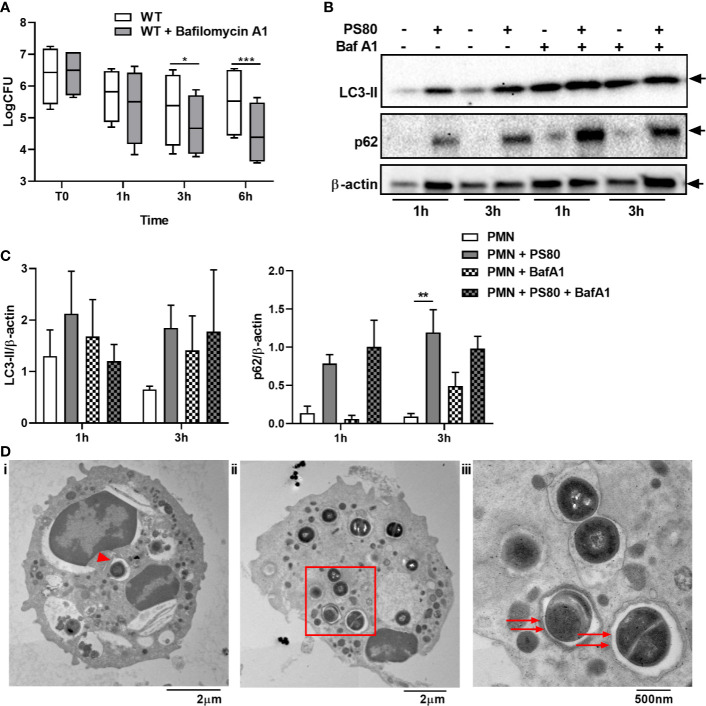
*Staphylococcus aureus* intracellular survival within PMN is associated with a disruption in autophagic flux. Primary human neutrophils were treated with Bafilomycin A1 (100 nM) or were left untreated and were then infected with pre-opsonized *S. aureus* PS80 (MoI 10) for 1 h. Following infection, PMN were treated with gentamicin (200 µg/ml) for the times indicated. **(A)** At each timepoint, PMN lysates were plated onto TSA and CFU enumerated. Data are expressed as Log CFU (n = 4 donors). Statistical analyses were performed using two-way ANOVA with Bonferroni post-tests. *P < 0.05; ***P < 0.001. At each timepoint, PMN protein lysates were probed for LC3 processing and p62 expression using Western immunoblotting **(B)** and analysed using densitometric analysis **(C)**. Data are expressed as protein expression normalized to β-actin control values for each sample ± SEM (n = 3–4 donors). Black arrows indicate the area of the blot used for densitometry. Statistical analyses were performed using two-way ANOVA with Bonferroni post-tests. **P < 0.01. At 3 h, infected PMN were imaged using transmission electron microscopy **(D)**. Whole, PS80-infected PMN showing phagophore formation indicated by red arrowhead (i) and double-membraned autophagosomes indicated by red arrows (ii) and inset (iii). Original magnification for Ci: 3000x, Cii: 2500x, Ciii: 8000x.

In order to further assess the effect of *S. aureus* on autophagic flux in PMN, PMN were pre-treated with the autophagy inhibitor Bafilomycin A1. Bafilomycin A1 treatment prevents autophagosome-lysosome fusion, therefore halting autophagic flux (32, 33). Intracellular survival of PS80 in Bafilomycin A1-treated PMN was significantly reduced at 3 and 6 h post-gentamicin treatment compared to untreated, infected PMN (**Figure 2A**), again indicating that a functioning autophagic pathway is required for intracellular survival. LC3 and p62 accumulation was determined in infected PMN compared to Bafilomycin A1 treated cells using Western immunoblotting. Increased levels of LC3 were observed in infected cells and Bafilomycin A1-treated cells compared to control PMN at 3 h post-gentamicin treatment (**Figures 2B, C**). No further increase in LC3 accumulation was evident in Bafilomycin A1-treated, infected cells compared to non-treated infected cells, suggesting that autophagosome-lysosome fusion has been blocked during intracellular survival. Accumulation of p62 was also increased in infected cells, Bafilomycin A1-treated cells and Bafilomycin A1-treated, infected cells at 3 h post-gentamicin treatment (**Figures 2B, C**), further indicating that normal autophagic flux has been altered. Taken together, these results indicate that *S. aureus* intracellular survival alters normal autophagic flux within PMN.

Although increased LC3-II levels are a strong indicator for an increase or block in autophagic flux, it can also be involved in non-canonical pathways that use autophagy machinery, such as LC3-associated phagocytosis (LAP). In order to confirm that *S. aureus* is using double-membraned autophagosomes as a niche, *S. aureus*-infected PMN were imaged using transmission electron microscopy at 3 h post-gentamicin treatment. Evidence of phagophore formation was observed (**Figure 2D**, i) and *S. aureus* dividing within double-membraned vesicles which are typically identified as autophagosomes (**Figure 2D**, ii, iii). Taken together, these data confirm that *S. aureus* uses the autophagy pathway in order to survive within human PMN during infection.

### *Staphylococcus aureus* Intracellular Survival Delays Apoptosis in Human PMN

**Figure 3:**
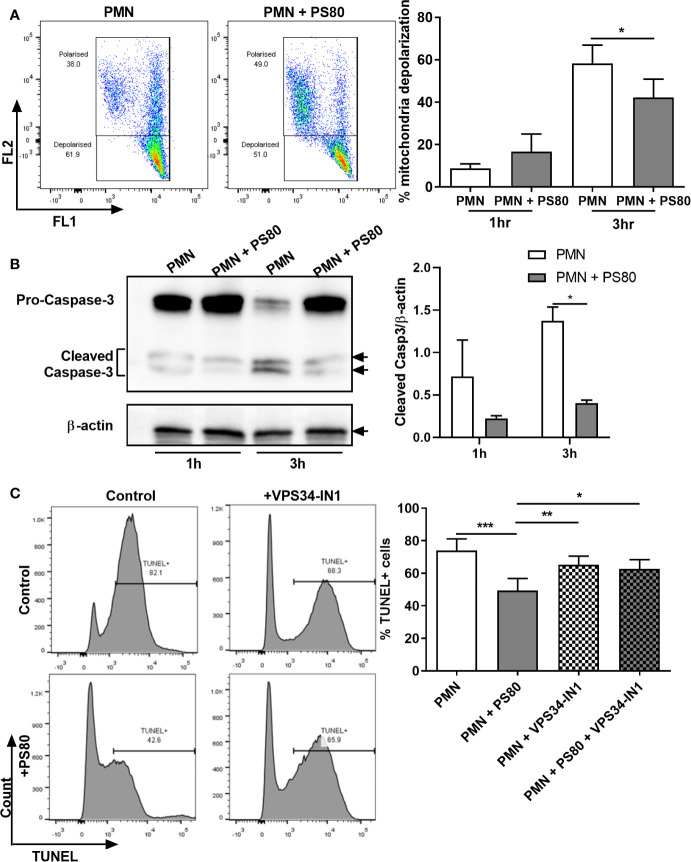
Hallmarks of apoptosis are delayed during *Staphylococcus aureus* intracellular survival. Primary human neutrophils were left untreated or were pre-treated with VPS34-IN1 (10 µM) and infected with pre-opsonized *S. aureus* PS80 (MoI 10) for 1 h. Following infection, PMN were treated with gentamicin (200 µg/ml) for the times indicated. **(A)** At each timepoint, PMN were stained with JC-1 dye to measure mitochondrial membrane depolarization and analyzed by flow cytometry (n = 3 donors). Representative FACS plots for JC-1 staining at 3 h are shown. Membrane depolarisation is characterized by a reduction of fluorescence in Fluorescent channel (FL) 2 and corresponding increase in FL1 fluorescence. Statistical analysis was performed using a paired t test. **(B)** PMN protein lysates were probed for caspase-3 expression and analysed using densitometric analysis. Data are expressed as protein expression normalized by β-actin control values for each sample ± SEM (n = 3 donors). Black arrows indicate the area of the blot used for densitometry. Statistical analysis was performed using two-way ANOVA with Bonferroni post-tests. *P < 0.05. **(C)** At 6 h, PMN were stained for DNA degradation using TUNEL staining and analysed by flow cytometry. Representative histograms for TUNEL-stained PMN for each treatment group are shown. Data are expressed as % TUNEL-positive cells ± SEM (n = 6 donors). Statistical analysis was performed using one-way ANOVA with Tukey post-tests. *P < 0.05; **P < 0.01; ***P < 0.001.

Our data indicate that *S. aureus* is using autophagosomes as a survival niche during infection in primary human PMN. We next assessed whether *S. aureus* could modulate PMN apoptosis in order to preserve its niche. Levels of early-stage apoptosis were determined by examining mitochondrial depolarization using JC-1 staining and caspase-3 cleavage by Western immunoblotting in *S. aureus*-infected PMN. At 3 h post-gentamicin treatment, infected PMN displayed significantly less mitochondrial depolarization (**Figure 3A**) and significantly lower levels of caspase-3 cleavage (**Figure 3B**) compared to uninfected controls, indicating a delay in caspase-mediated apoptosis during intracellular survival. The effects of intracellular survival on late-stage apoptosis were then determined using TUNEL staining. At 6 h post-gentamicin treatment, a mean of 73.9% of untreated PMN were TUNEL-positive whereas infected PMN were significantly less apoptotic (**Figure 3C**, mean value: 49.4%), confirming that apoptosis is delayed during intracellular survival.

In order to determine if the observed changes in apoptosis were associated with the presence of intracellular *S. aureus* as a result of subverting the autophagy pathway, apoptosis levels were determined in infected PMN following pre-treatment with VPS34-IN1. After 6 h, VPS34-IN1-treated *S. aureus*-infected PMN had significantly more TUNEL-positive staining than infected, untreated PMN (**Figure 3C**, mean value: 62.7%). PMN treated with VPS34-IN1 alone displayed similar levels of TUNEL staining as VPS34-IN1-treated *S. aureus*-infected PMN and PMN alone (mean value: 65.2%). This indicates that the reduction in apoptosis observed in infected PMN was reversed by inhibiting autophagy-mediated intracellular survival. Although not statistically significant, a similar trend was evident for an increase in caspase-3 cleavage (**Figure S2A**) but less so for mitochondrial depolarization (**Figure S2B**) in infected, VPS34-IN1-treated PMN at 3 h post-gentamicin treatment. Taken together, these results indicate that apoptosis is delayed during *S. aureus* intracellular survival. Furthermore, blocking autophagy may partially relieve the inhibitory effect caused by *S. aureus* on the apoptosis pathway.

### *Staphylococcus aureus* Intracellular Survival Is Associated With an Anti-Apoptotic Phenotype and Activation of the p53/DRAM Pathway

**Figure 4:**
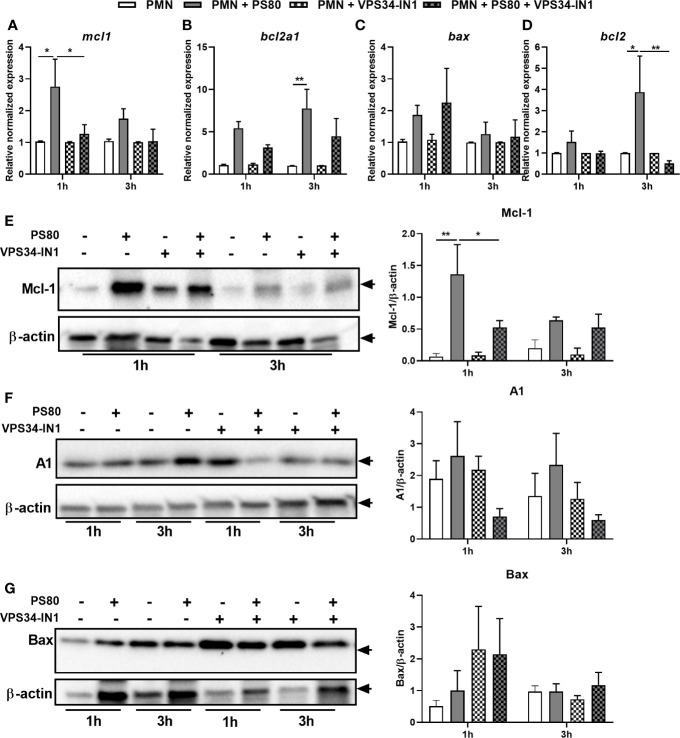
*Staphylococcus aureus* intracellular survival is associated with changes in expression of apoptotic factors and an anti-apoptotic phenotype. Primary human neutrophils were left untreated or were pre-treated with VPS34-IN1 (10 µM) and were then infected with pre-opsonized *S. aureus* PS80 (MoI 10) for 1 h. Following infection, PMN were treated with gentamicin (200 µg/ml) for the times indicated. RNA was extracted and gene expression levels of *mcl1* **(A)**, *bcl2a1* **(B)**, *bax* **(C)**, and *bcl2* **(D)** assessed using quantitative RT-PCR at 1 and 3 h post-gentamicin treatment. Gene expression is plotted relative to gene expression in control PMN after normalization to 18s RNA ± SEM (n = 3–4 donors). Statistical analysis was performed using two-way ANOVA with Tukey post-tests. *P < 0.05; **P < 0.01. PMN protein lysates were probed for Mcl-1 **(E)**, A1/Bfl-1 **(F)**, and Bax **(G)** expression and analysed using densitometric analysis. Data are expressed as protein expression normalized by β-actin control values for each sample ± SEM (n = 3–4 donors). Black arrows indicate the area of the blot used for densitometry. Statistical analysis was performed using two-way ANOVA with Bonferroni post-tests. *P < 0.05; **P < 0.01.

The delay in apoptosis observed in infected PMN coupled with the incomplete restoration of apoptosis in inhibitor-treated, infected PMN suggests a complex apoptotic phenotype is occurring during *S. aureus* intracellular survival. In order to further characterize this phenotype, expression of apoptotic genes involved in the intrinsic apoptotic pathway was determined using RT-PCR and corresponding protein expression was determined using Western immunoblotting. Expression of the anti-apoptotic factor *mcl1* was significantly increased at 1 h post-gentamicin treatment (**Figure 4A**) with a similar significant increase in protein expression evident at 1 h (**Figure 4E**). Gene expression of anti-apoptotic factors *bcl2a1* and *bcl2* were significantly increased after 3 h (**Figures 4B, D**). A similar trend towards higher protein level of A1/Bfl-1 was observed (**Figure 4F**); however, protein levels of Bcl-2 could not be detected in PMN under any conditions. Neither gene expression nor protein expression of pro-apoptotic factor *bax* was significantly increased in infected PMN at either timepoint (**Figures 4C, G**), confirming that intracellular survival elicits an anti-apoptotic phenotype in PMN.

**Figure 5:**
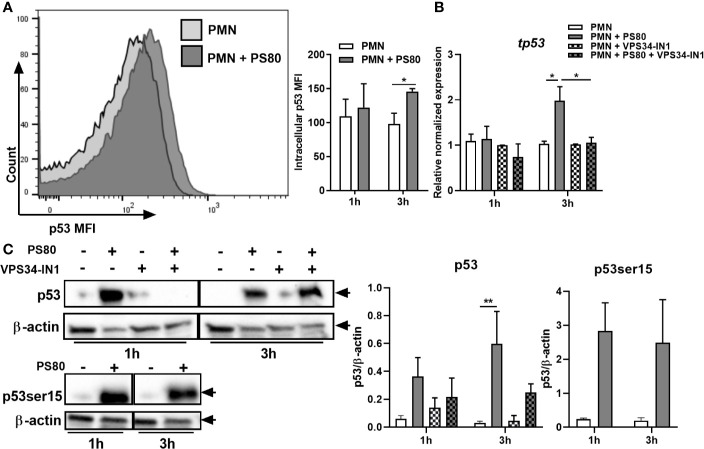
The p53/DRAM pathway is activated during *Staphylococcus aureus* intracellular survival. Primary human neutrophils were left untreated or were pre-treated with VPS34-IN1 (10 µM) and were then infected with pre-opsonized *S. aureus* PS80 (MoI 10) for 1 h. Following infection, PMN were treated with gentamicin (200 µg/ml) for the times indicated. At each timepoint, intracellular staining for p53 was carried out and analysed by flow cytometry. **(A)** Representative FACS plot showing p53 intracellular staining at 3 h and MFI values of nuclear p53. RNA was extracted and gene expression levels of **(B)** *tp53* was assessed using quantitative RT-PCR. Gene expression is plotted relative to gene expression in control PMN after normalization to 18s RNA ± SEM (n = 3–4 donors). Statistical analysis was performed using two-way ANOVA. *P < 0.05. **(C)** PMN protein lysates were probed for total p53 and p53ser15 expression and analysed using densitometric analysis. Data are expressed as protein expression normalized by β-actin control values for each sample ± SEM (n = 3–4 donors). Black arrows indicate the area of the blot used for densitometry. Statistical analysis was performed using two-way ANOVA with Bonferroni post-tests. **P < 0.01.

These results indicate that the intrinsic apoptotic pathway is delayed during intracellular survival due to the upregulation of anti-apoptotic factors. Therefore, we next looked at transcriptional regulation of these factors by determining intracellular levels of the cell-cycle transcription factor p53. p53 is a well-defined positive regulator of the intrinsic apoptotic pathway; however, when localized to the nucleus, p53 can positively regulate autophagy in response to cellular stress (34, 35). Intracellular levels of p53 were determined in infected PMN using flow cytometry and expression of *tp53* was confirmed using RT-PCR and Western immunoblotting. At 3 h post-gentamicin treatment, intracellular levels of p53 were significantly higher in infected PMN compared to controls (**Figure 5A**). Gene and protein expression of p53 (**Figures 5B, C**) was significantly increased at 3 h post-gentamicin treatment. In order to confirm the intracellular location of p53, expression levels of p53 phosphorylated at Serine 15 (p53ser15) were determined. Phosphorylation of p53 at serine 15 has been previously shown to inhibit a nuclear export signal at the amino terminal of p53 (36); therefore, high levels of p53ser15 may indicate retention of p53 in the nucleus. Protein levels of p53ser15 were determined by Western immunoblotting (**Figure 5C**). High levels of p53ser15 were detected at 1 and 3 h post-gentamicin treatment compared to control PMN, suggesting that p53 is being retained in the nucleus during intracellular survival.

**Figure 6:**
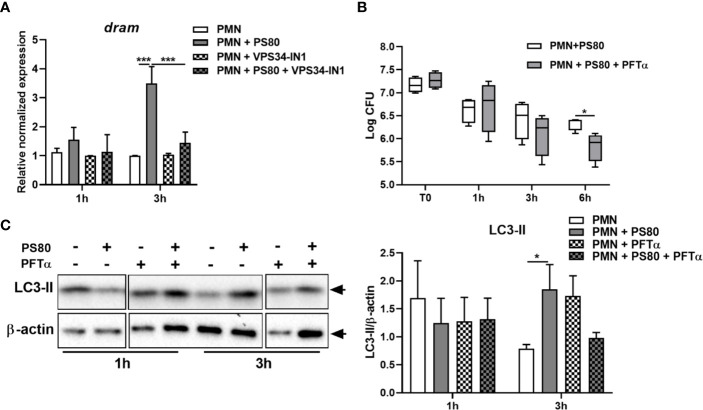
Inhibition of p53 reduces *Staphylococcus aureus* intracellular survival in neutrophils. **(A)** Primary human neutrophils were left untreated or were pre-treated with VPS34-IN1 (10 µM) and were then infected with pre-opsonized *S. aureus* PS80 (MoI 10) for 1 h. Following infection, PMN were treated with gentamicin (200 µg/ml) for the times indicated. RNA was extracted and gene expression levels of *dram* assessed using quantitative RT-PCR. Gene expression is plotted relative to gene expression in control PMN after normalization to 18s RNA ± SEM (n = 3–4 donors). Statistical analysis was performed using two-way ANOVA. ***P < 0.001. **(B)** Primary human neutrophils were treated with Pifithrin-α (30μM) or were left untreated and were then infected with pre-opsonized *S. aureus* PS80 (MoI 10) for 1 h. Following infection, PMN were treated with gentamicin (200 µg/ml) for the times indicated. At each timepoint, PMN lysates were plated onto TSA and CFU enumerated. Data are expressed as Log CFU (n = 4 donors). Statistical analyses were performed using two-way ANOVA with Bonferroni post-tests. *P < 0.05. **(C)** PMN protein lysates were probed for LC3 processing using Western immunoblotting and analysed using densitometric analysis. Data are expressed as protein expression normalized to β-actin control values for each sample ± SEM (n = 3 donors). Black arrows indicate the area of the blot used for densitometry. Statistical analyses were performed using two-way ANOVA with Bonferroni post-tests. *P < 0.05.

Nuclear p53 induces transcription of the pro-autophagic membrane protein Damage-Regulated Autophagy Monitor (DRAM). At 3 h post-gentamicin treatment, gene expression of *dram* (**Figure 6A**) was significantly increased in *S. aureus*-infected PMN. Although detection of DRAM at the protein level proved challenging, there was also evidence of increased DRAM protein expression at 3 h post-gentamicin treatment (**Figure S3**). This confirms that p53-activated gene transcription is occurring during intracellular survival, and suggests that autophagy-mediated intracellular survival is activating the p53/DRAM stress response pathway.

To determine whether the changes in apoptotic gene expression are directly influenced by autophagy-induced intracellular survival, gene and protein expression of Mcl-1, Bcl-2, A1/Bfl-1, and Bax was assessed in infected PMN pre-treated with VPS34-IN1. Autophagy inhibition resulted in significantly reduced expression of *mcl1* and *bcl2* at 1 and 3 h post-gentamicin treatment, respectively compared to untreated infected controls (**Figures 4A, D**). Mcl-1 protein expression in infected PMN was also reduced significantly after VPS34-IN1 treatment 1 h post-gentamicin treatment (**Figure 4E**). Gene expression of *bcl2a1* and *bax* were not significantly affected (**Figures 4C, E**) although when compared to infected PMN, A1/Bfl-1 protein expression appeared to decrease in infected PMN after VPS34-IN1 treatment, albeit not significantly (**Figure 4F**). Similarly, Bax protein expression appeared to increase after VPS34-IN1 treatment 1 h post-gentamicin treatment, but not significantly (**Figure 4G**). These results indicate that autophagy inhibition elicits a partial restoration of the apoptotic state in PMN during intracellular survival.

The effect of autophagy inhibition on the expression of *tp53* and *dram* was also assessed. Expression of *tp53* and *dram* were both significantly decreased in the presence of VPS34-IN1 at 3 h post-gentamicin treatment (**Figures 5B** and **6A**) and protein expression of p53 appeared decreased at 3 h post-gentamicin treatment compared to untreated, infected PMN (**Figure 5C**), indicating that the p53/DRAM pathway is also directly influenced by autophagy-mediated intracellular survival. In order to further assess the effect of the p53 pathway on intracellular survival, PMN were pre-treated with a p53 inhibitor, Pifithrin-α (PFTα) and intracellular survival was assessed. Intracellular survival of *S. aureus* in PFTα-treated PMN was significantly reduced at 6 h post-gentamicin treatment (**Figure 6B**). Gene expression of *tp53* and *dram* were assessed at 1 and 3 h post-gentamicin treatment and were confirmed to be lower in PFTα-treated cells (**Figure S4**). Autophagic flux was assessed in the presence of PFTα by determining the levels of LC3-II in infected PMN at 1 and 3 h post-gentamicin treatment. After 1 h gentamicin treatment, both untreated and PFTα-treated, infected PMN had similar protein levels of LC3-II (**Figure 6C**). After 3 h gentamicin-treatment, LC3-II protein levels were higher in infected PMN compared to uninfected PMN, whereas in PFTα-treated, infected PMN, LC3-II levels were lower compared to the corresponding uninfected control (**Figure 6C**) suggesting that autophagic flux has been restored. Together, these data indicate that the p53 pathway plays an important role in autophagy-mediated intracellular survival of *S. aureus*.

Overall, these data indicate that autophagy-mediated intracellular survival activates the p53/DRAM stress response while simultaneously promoting the expression of several anti-apoptotic factors which may counteract a pro-apoptotic role for p53. This promotes an anti-apoptotic, pro-autophagic phenotype in human PMN, prolonging the intracellular niche for *S. aureus*.

## Discussion

*Staphylococcus aureus* bloodstream infection is a significant cause of morbidity and mortality worldwide (1, 2, 37). Persistence of bacteraemia and metastatic infection is associated with failure to eradicate the source of infection, suggesting that an intracellular reservoir for *S. aureus* exists (4). Due to the prevalence of *S. aureus* antibiotic resistance, new host-directed therapies are required where antibiotics are no longer effective. A detailed knowledge of how *S. aureus* manipulates the innate immune response and survives during bacteraemia is needed in order to develop these therapies. In this study, we show that *S. aureus* survives intracellularly within primary human PMN by manipulating the autophagy pathway to establish an intracellular niche, while simultaneously inhibiting the normal apoptotic pathway.

Previous studies have identified the autophagy pathway as a mechanism of intracellular survival for *S. aureus* during infection in several cell types *in vitro* (17, 18, 38), while subversion of autophagy within PMN for bacterial survival has previously been reported for *E. coli* (39). Interestingly, a recent study reported that *S. aureus* can survive within LC3-decorated phagosomes in PMN in larval zebrafish using the non-canonical form of autophagy, LAP (40). Using primary human PMN we observed *S. aureus* present in double-membraned autophagosomes using TEM which is a characteristic of canonical selective autophagy rather than LAP. However, a role for LAP in human PMN requires further investigation. Our study identifies a novel role for autophagy in *S. aureus* survival within a primary human professional phagocyte. A disruption in autophagic flux was evident in infected PMN and intracellular survival decreased significantly when autophagy was inhibited. These results provide strong evidence of manipulation of the autophagic pathway in *S. aureus* in order to survive intracellularly.

The changes in autophagy reported here are accompanied by a decrease in apoptosis in human PMN harboring *S. aureus*. These results reflect changes to the intrinsic apoptotic pathway that may prolong the PMN life cycle and therefore the intracellular niche for *S. aureus*. In previous studies examining changes in the PMN life cycle during *S. aureus* infection *in vitro*, PMN were reported to display regular markers of apoptosis but also exhibited signs of a dysregulated apoptosis phenotype (25). Other studies illustrated a delay in PMN apoptosis during *S. aureus* infection (24), and the apoptotic fate of PMN during *S. aureus* infection was shown to depend on multiplicity of infection (41). These conflicting accounts of the changes to PMN lifespan during *S. aureus* infection, coupled with our data, confirm that the apoptotic fate of PMN is context-dependent. Several *in vivo* studies have demonstrated rapid dissemination of *S. aureus* to secondary infection sites within hours of initial infection, and PMN have been implicated as potential mediators (16, 42). Furthermore, PMN isolated from *S. aureus* infection sites have been shown to contain viable bacteria capable of re-infecting a naïve host (8). Therefore, any delay, even briefly, in PMN apoptosis would give *S. aureus* a survival advantage for long enough to potentially proliferate and disseminate, supporting the “Trojan horse” theory of *S. aureus* immune evasion.

*S. aureus* survival in PMN is significantly reduced using an agr-deficient mutant. Furthermore, there was no further decrease in survival in Δagr-infected, VPS34-IN1-treated PMN, indicating that an agr-specific factor is important for autophagy-mediated intracellular survival. Previously, autophagy-dependent intracellular survival in HeLa cells was shown to depend on a factor controlled by the Agr operon (17). Subsequent work on *S. aureus* subversion of the autophagic pathway, has highlighted the role of the agr-regulated secreted toxin α-haemolysin (Hla) in driving the production of autophagosomes which facilitated bacterial replication in non-professional phagocytes (38). Furthermore, Hla expression is also essential for *S. aureus* phagosomal escape from cystic fibrosis epithelial cells (43). However, one study analysing the role of autophagy in *S. aureus* infection *in vivo* demonstrated that the autophagic pathway conferred protection against Hla-mediated toxic effects in a murine model of *S. aureus* systemic infection (44). While autophagy-deficient mice were more susceptible to lethality during *S. aureus* infection, challenge with a Hla-deficient strain led to increased survival. This suggests that the role of Hla in autophagy-mediated intracellular survival is not a straightforward one. Moreover, several studies have reported that Hla can induce cell death in the form of apoptosis and necrosis (45–48). The mechanism of Hla-induced cell death is unclear; some studies report that Hla induces caspase-independent cell death (47) while others report the involvement of Caspase-2, 3 and 8 (45, 46). Our data further implicates the involvement of an agr-specific factor in manipulating the autophagy network in human PMN but whether it is Hla has yet to be determined.

The transcription factor p53 plays a regulatory role in apoptosis and is activated as part of the host response to cellular stress, causing cell cycle arrest and initiating programmed cell death (49, 50). Our results indicate that p53 is activated following autophagy-mediated intracellular survival. This is in contrast to other studies that indicate that p53 is an autophagy inducer (34, 50, 51). However, a recent study has shown that the VPS34/Beclin-1 complex can act as a regulator of p53 due to its regulatory effect on the ubiquitin-specific protease, USP10, which mediates p53 deubiquitination (52). By regulating USP10 activity, the VPS34 autophagy activation complex can control p53 degradation, highlighting a regulatory relationship between the VPS34 complex and p53. In our model, p53 is transcriptionally active as evidenced by an increased expression of *dram*. However, the pro-apoptotic effects of p53 are not evident in our model as we see no significant change in the gene or protein expression of *bax* which is also transcriptionally activated by p53. As well as *dram*, nuclear p53 can drive the transcriptional activation of several autophagy related genes such as *ulk1* and *atg7* (51), and DRAM can directly mediate p53-induced autophagy (34). Expression of *dram* may be re-enforcing the pro-autophagic state in our model. Importantly, we demonstrate that p53 expression can promote autophagy-dependent intracellular survival since p53 inhibition using PFTα resulted in lower intracellular CFU coupled with increased autophagic flux. Decreased *tp53*/*dram* expression was observed after autophagy inhibition, confirming that the pro-autophagic effects of the p53/DRAM pathway are a direct result of *S. aureus*-induced autophagy.

Although DRAM can also drive p53-mediated apoptosis (34), it does not appear to be involved in promoting apoptosis during *S. aureus* intracellular survival. Instead, we observed a striking induction of anti-apoptotic factors. Our results suggest that gene expression of Mcl-1 and Bcl-2 are activated as a direct result of autophagy-mediated intracellular survival since blocking autophagy in PMN inhibited the gene expression and lead to a reduction in protein expression of both. Mcl-1 and Bcl-2 can prevent Fas-mediated PMN apoptosis (53) and both are upregulated in PMN from patients with sepsis (54, 55). Furthermore, Mcl-1 and Bcl-2 transcription could protect macrophages from staurosporine-induced apoptosis during *S. aureus* infection (56). Although we saw an increase in Bcl2 gene expression, we could not confirm Bcl-2 protein expression using Western immunoblotting. The absence of Bcl-2 protein expression in mature neutrophils has been reported previously (57). Expression of *bcl2a1*, which encodes A1/Bfl-1, was significantly upregulated during intracellular survival with a similar increase in protein expression. However, *bcl2a1* expression was not significantly affected by autophagy inhibition and therefore, A1/Bfl-1 may still be exerting some anti-apoptotic effects. This consistent expression of *bcl2a1* may account for why levels of mitochondrial depolarization and caspase-3 cleavage are not completely restored in infected, VPS34-IN1-treated PMN. A1/Bfl-1 has previously been implicated in delaying PMN apoptosis during *Anaplasma phagocytophilum* infection by maintaining high mitochondrial membrane potential and inhibiting caspase-3 activity (58). A1/Bfl-1 has been found to actually bind pro-caspase-3 and prevents its activation in immortalized motor neurons (59) and A1/Bfl-1 has been shown to overcome p53-mediated apoptosis (60). In our study, it seems that any pro-apoptotic effect of p53 is overwhelmed by a potent anti-apoptotic phenotype both linked to autophagy-mediated intracellular survival through Mcl-1 expression, and an autophagy-independent mechanism through A1/Bfl-1 expression. This may be enough to transiently maintain an anti-apoptotic status that is beneficial for intracellular survival. Further study may determine whether this is specifically mediated by a bacterial factor.

**Figure 7:**
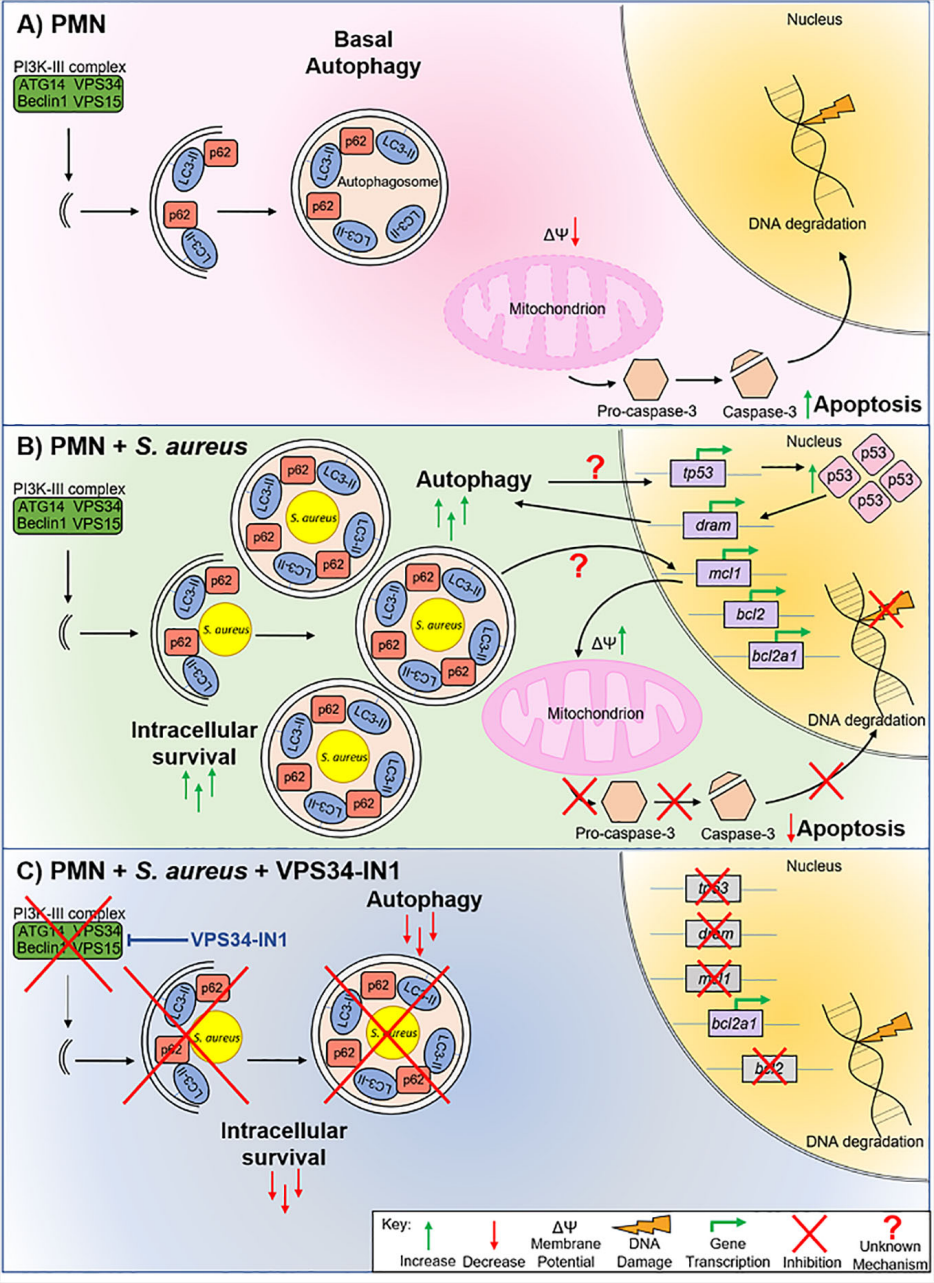
Model of *S. aureus* intracellular survival in human PMN. Uninfected PMN **(A)** undergo basal autophagy. Programmed cell death also occurs *via* mitochondrial depolarization, caspase-3 cleavage and eventual DNA degradation. PMN harboring *S. aureus* **(B)** undergo a higher rate of autophagy. *S. aureus* is encapsulated in autophagosomes which facilitates intracellular survival. Autophagy-mediated intracellular survival triggers *tp53* transcription and p53 protein levels increase in the nucleus. Transcription of pro-autophagic factor *dram*, as well as anti-apoptotic factors *mcl1*, *bcl2* and *bcl2a1*, is increased. As a result of the expression of these anti-apoptotic factors, mitochondrial membrane depolarization is reduced, caspase-3 cleavage is inhibited, and DNA degradation is reduced, indicating a lower rate of apoptosis during intracellular survival. Blocking autophagy using VPS34-IN1 **(C)** decreases intracellular survival of *S. aureus* by removing the availability of an intracellular niche. Blocking autophagy also reduces transcription of *tp53*, *dram*, *mcl1*, and *bcl2* suggesting that crosstalk between the autophagy and apoptotic pathways occurs at this level. DNA degradation is returned to basal levels suggesting that blocking autophagy-mediated intracellular survival partially restores apoptosis in PMN.

Based on our findings, we propose the following model: Under basal conditions, homeostatic autophagy occurs in PMN and PMN undergo spontaneous apoptosis as part of their normal lifespan (**Figure 7A**). After *S. aureus* exposure (**Figure 7B**), PMN harboring *S. aureus* accumulate autophagosomes which act as an intracellular niche. The p53 pathway is activated and p53 accumulates in the nucleus. Nuclear p53 drives transcription of *dram*, reinforcing a pro-autophagic phenotype. Transcription of anti-apoptotic factors *bcl2, mcl1* and *bcl2a1* is activated by autophagy-mediated intracellular survival *via* an unknown mechanism. The expression of these factors appears to be enough to overcome any pro-apoptotic effects of p53, leading to a slower rate of apoptosis as evidenced by inhibition of mitochondrial depolarization, a delay in caspase-3 cleavage and lower levels of DNA degradation. When autophagy is blocked using VPS34-IN1, autophagy-mediated intracellular survival is inhibited (**Figure 7C**). The transcription of *tp53, dram, mcl1* and *bcl2*, but not *bcl2a1* and *bax*, is inhibited following autophagy inhibition, leading to a partially restored apoptotic pathway in PMN.

The findings in this study illustrate that *S. aureus* infection in phagocytes represents a complex host-pathogen interaction. The changes identified in host pathways during intracellular survival present possible therapeutic targets as an additive treatment for *S. aureus* infection. Blanket autophagy inhibition should, however, be pursued with caution as this may lead to undesirable downstream effects for the host. However, inhibitors of anti-apoptotic factors such as Mcl-1, already in development in cancer therapy (61), may restore an appropriate apoptotic pathway in PMN and disrupt the intracellular niche for *S. aureus*. An intricate knowledge of the mechanism of manipulation of both pathways during *S. aureus* infection is crucial prior to providing more targeted treatment.

## Data Availability Statement

The raw data supporting the conclusions of this article will be made available by the authors, without undue reservation.

## Ethics Statement

The studies involving human participants were reviewed and approved by School of Biochemistry and Immunology Research Ethics Committee, Trinity College Dublin. The patients/participants provided their written informed consent to participate in this study.

## Author Contributions

MM, KO’K, and RM conceived and designed experiments. MM, KO’K, EO’B, and NL performed experiments and collected data. MM, EO’B, and EV analyzed the data. MM and RM wrote the manuscript. All authors contributed to the article and approved the submitted version.

## Funding

This work was supported by a Science Foundation Ireland Investigator Award (15/IA/3041) and a Wellcome Investigator Award (202846/Z/16/Z) to RM.

## Conflict of Interest

The authors declare that the research was conducted in the absence of any commercial or financial relationships that could be construed as a potential conflict of interest.

## Acknowledgments

We thank Andrew Edwards from Imperial College London for the gift of USA300 LAC ΔagrC and its parental strain. We also thank James Murray for his input on the autophagy studies.

## Supplementary Material

Supplementary Figure 1 VPS34-IN1 has no effect on phagocytic uptake of *Staphylococcus aureus* in human neutrophils. Primary human neutrophils were pre-treated with VPS34-IN1 (10 µM) or DMSO for 30 min prior to infection with pre-opsonized *S. aureus* PS80-GFP (MoI 10) for 1 h. Following infection, PMN were treated with gentamicin (200 µg/ml) for 30 min. Cells were fixed and analyzed by flow cytometry. Data are expressed as mean %GFP-positive cells ± SEM (n = 2 donors).

Supplementary Figure 2 VPS34-IN1 treatment may partially restore early apoptosis in PMN harboring *S. aureus*. Primary human neutrophils were left untreated or were pre-treated with VPS34-IN1 (10 µM) and were then infected with pre-opsonized *S. aureus* PS80 (MoI 10) for 1 h. Following infection, PMN were treated with gentamicin (200 µg/ml) for the times indicated. (A) PMN protein lysates were probed for caspase-3 cleavage and analysed using densitometric analysis. Data are expressed as protein expression normalized by β-actin control values for each sample ± SEM (n = 3 donors). (B) PMN were stained with JC-1 dye to measure mitochondrial membrane depolarization and analyzed by flow cytometry (n = 3 donors). Representative FACS plots for JC-1 staining at 3 h. Membrane depolarisation is characterized by a reduction of fluorescence in Fluorescent channel (FL) 2 and corresponding increase in FL1 fluorescence.

Supplementary Figure 3 DRAM protein expression during *Staphylococcus aureus* intracellular survival in neutrophils. Primary human neutrophils were infected with pre-opsonized *S. aureus* PS80 (MoI 10) for 1 h. Following infection, PMN were treated with gentamicin (200 µg/ml) for 3 h. PMN protein lysates were probed for DRAM expression and analysed using densitometric analysis. Data are expressed as protein expression normalized by β-actin control values for each sample ± SEM (n = 2 donors). Two representative blots are shown. Black arrows indicate the area of the blot used for densitometry.

Supplementary Figure 4
*tp53* and *dram* expression after Pifithrin-α treatment during *Staphylococcus aureus* intracellular survival in neutrophils. Primary human neutrophils were treated with Pifithrin-α (30μM) or were left untreated and were then infected with pre-opsonized *S. aureus* PS80 (MoI 10) for 1 h. Following infection, PMN were treated with gentamicin (200 µg/ml) for the times indicated. RNA was extracted and gene expression levels of (A) *tp53* and (B) *dram* were assessed using quantitative RT-PCR. Gene expression is plotted relative to gene expression in control PMN after normalization to 18s RNA ± SEM (n = 2 donors).
