# Supplementary figures and images for: Manipulation of Autophagy and Apoptosis Facilitates Intracellular Survival of Staphylococcus aureus in Human Neutrophils
Source: Front Immunol. 2020 Nov 11;11:565545. doi: 10.3389/fimmu.2020.565545 (PMC7686353; doi:10.3389/fimmu.2020.565545)

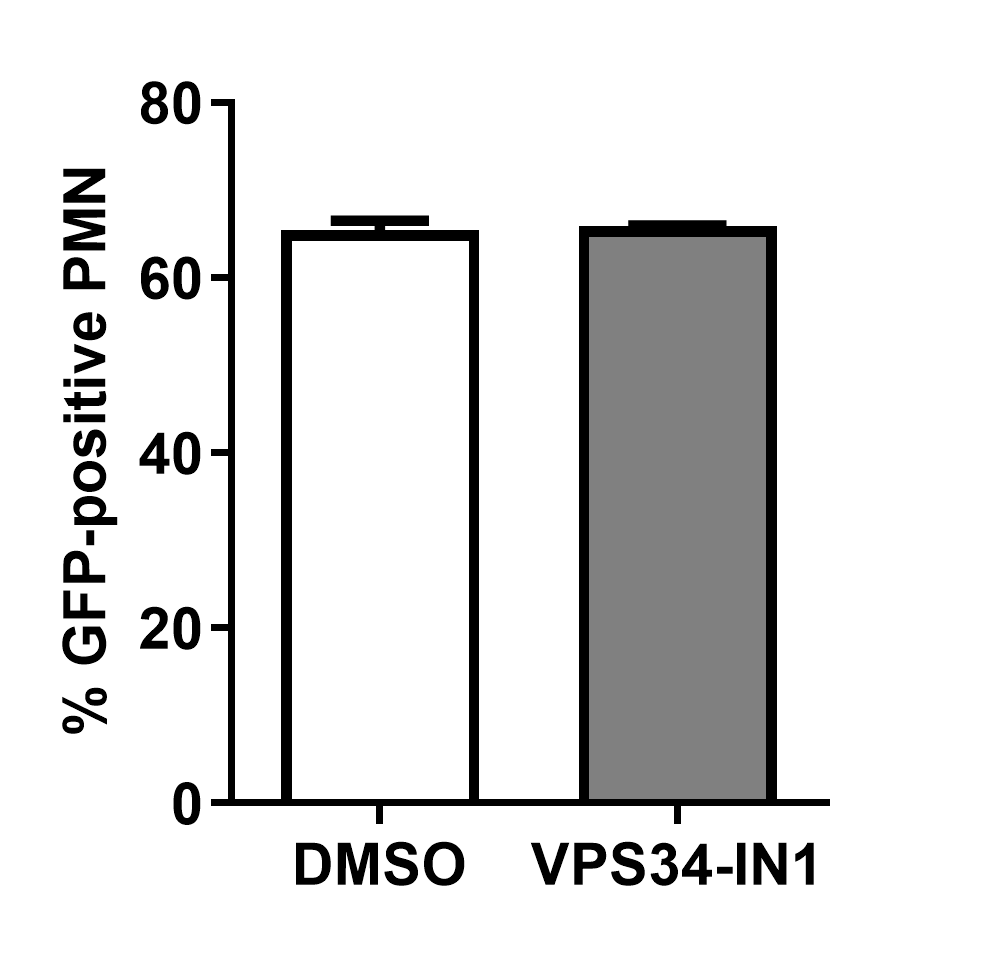

Supplement: Supplementary Figure 1 — VPS34-IN1 has no effect on phagocytic uptake of Staphylococcus aureus in human neutrophils. Primary human neutrophils were pre-treated with VPS34-IN1 (10 µM) or DMSO for 30 min prior to infection with pre-opsonized S. aureus PS80-GFP (MoI 10) for 1 h. Following infection, PMN were treated with gentamicin (200 µg/ml) for 30 min. Cells were fixed and analyzed by flow cytometry. Data are expressed as mean %GFP-positive cells ± SEM (n = 2 donors). [file Image_1.tif]

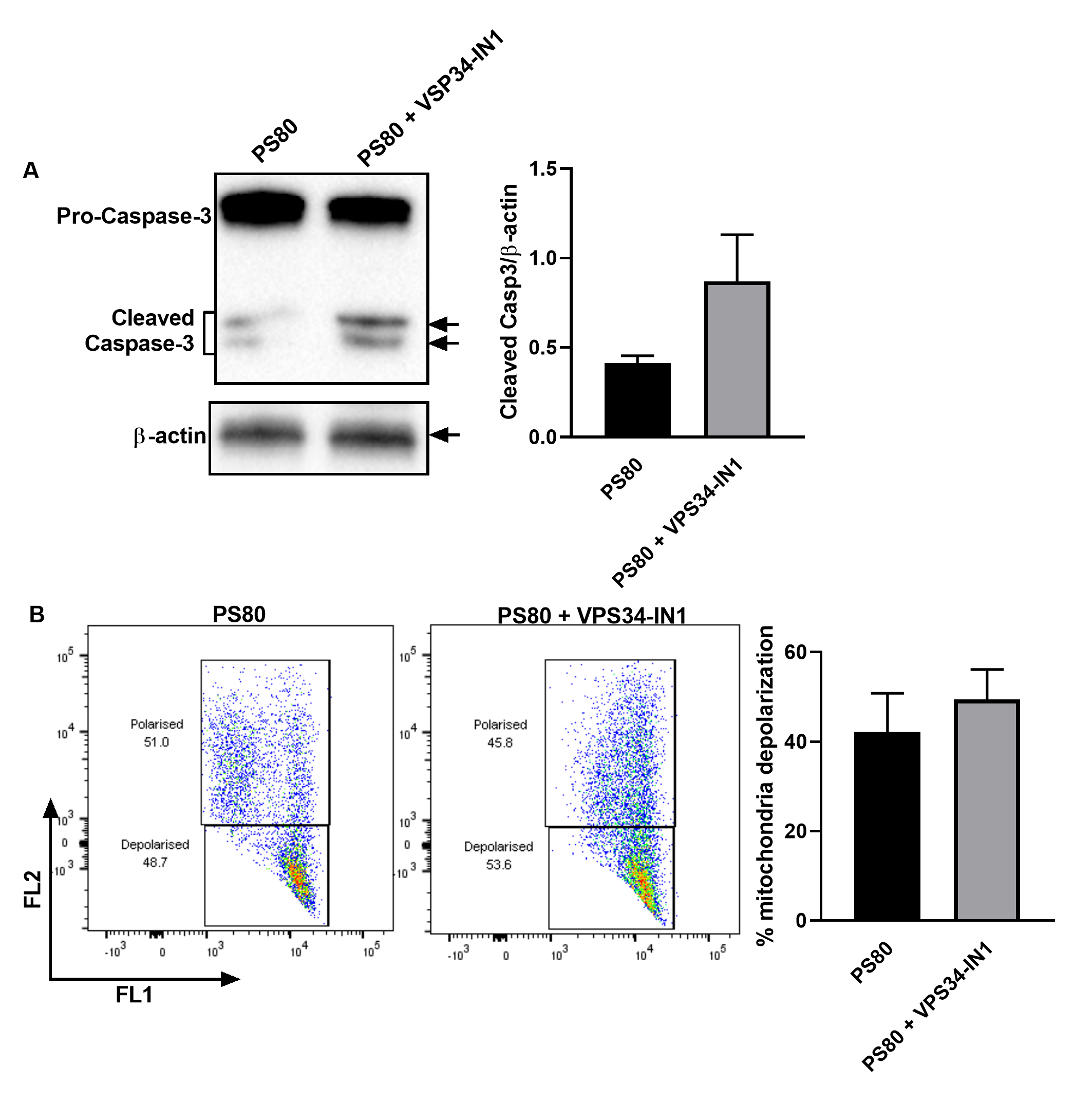

Supplement: Supplementary Figure 2 — VPS34-IN1 treatment may partially restore early apoptosis in PMN harboring S. aureus. Primary human neutrophils were left untreated or were pre-treated with VPS34-IN1 (10 µM) and were then infected with pre-opsonized S. aureus PS80 (MoI 10) for 1 h. Following infection, PMN were treated with gentamicin (200 µg/ml) for the times indicated. (A) PMN protein lysates were probed for caspase-3 cleavage and analysed using densitometric analysis. Data are expressed as protein expression normalized by β-actin control values for each sample ± SEM (n = 3 donors). (B) PMN were stained with JC-1 dye to measure mitochondrial membrane depolarization and analyzed by flow cytometry (n = 3 donors). Representative FACS plots for JC-1 staining at 3 h. Membrane depolarisation is characterized by a reduction of fluorescence in Fluorescent channel (FL) 2 and corresponding increase in FL1 fluorescence. [file Image_2.tif]

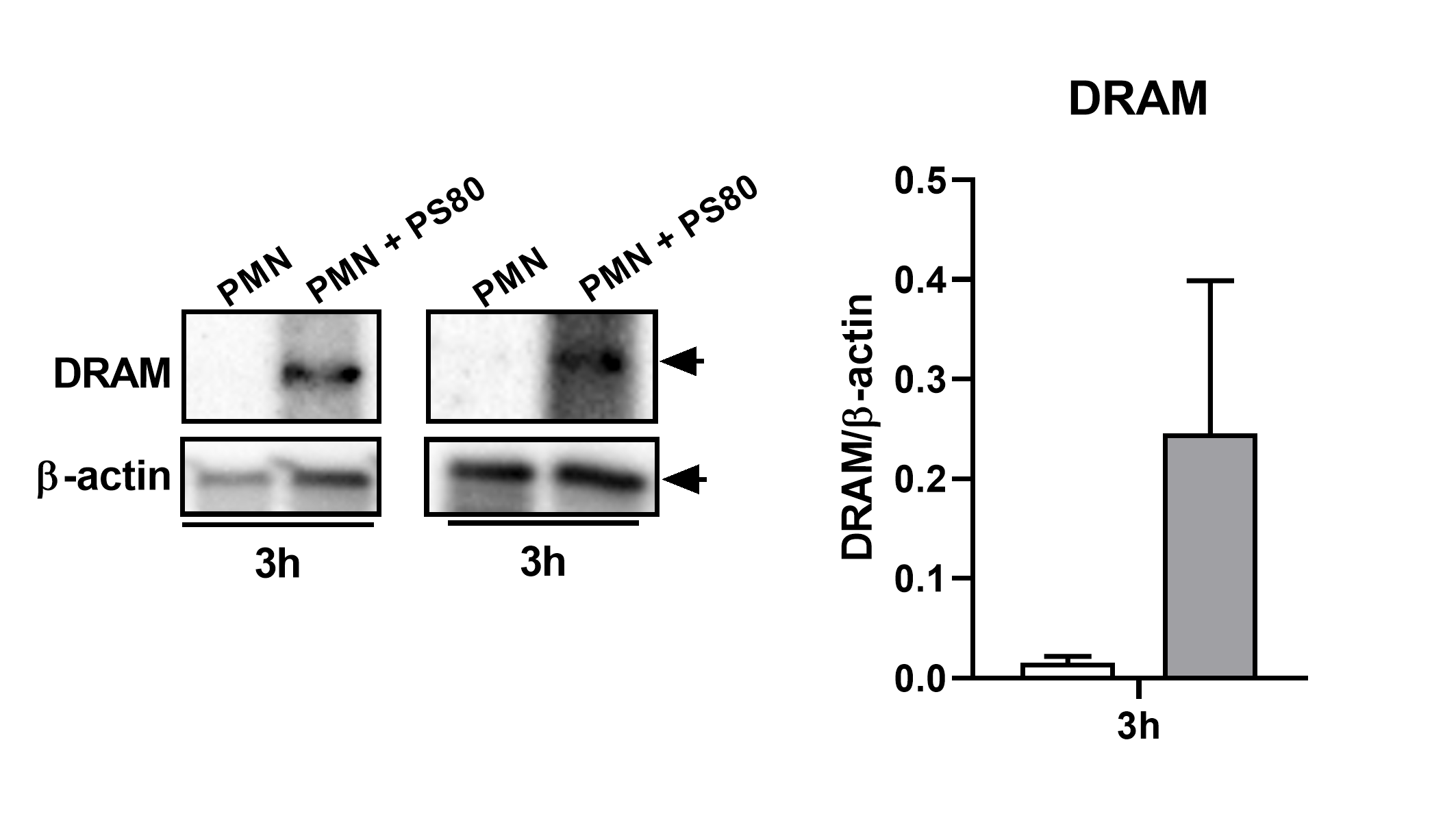

Supplement: Supplementary Figure 3 — DRAM protein expression during Staphylococcus aureus intracellular survival in neutrophils. Primary human neutrophils were infected with pre-opsonized S. aureus PS80 (MoI 10) for 1 h. Following infection, PMN were treated with gentamicin (200 µg/ml) for 3 h. PMN protein lysates were probed for DRAM expression and analysed using densitometric analysis. Data are expressed as protein expression normalized by β-actin control values for each sample ± SEM (n = 2 donors). Two representative blots are shown. Black arrows indicate the area of the blot used for densitometry. [file Image_3.tif]

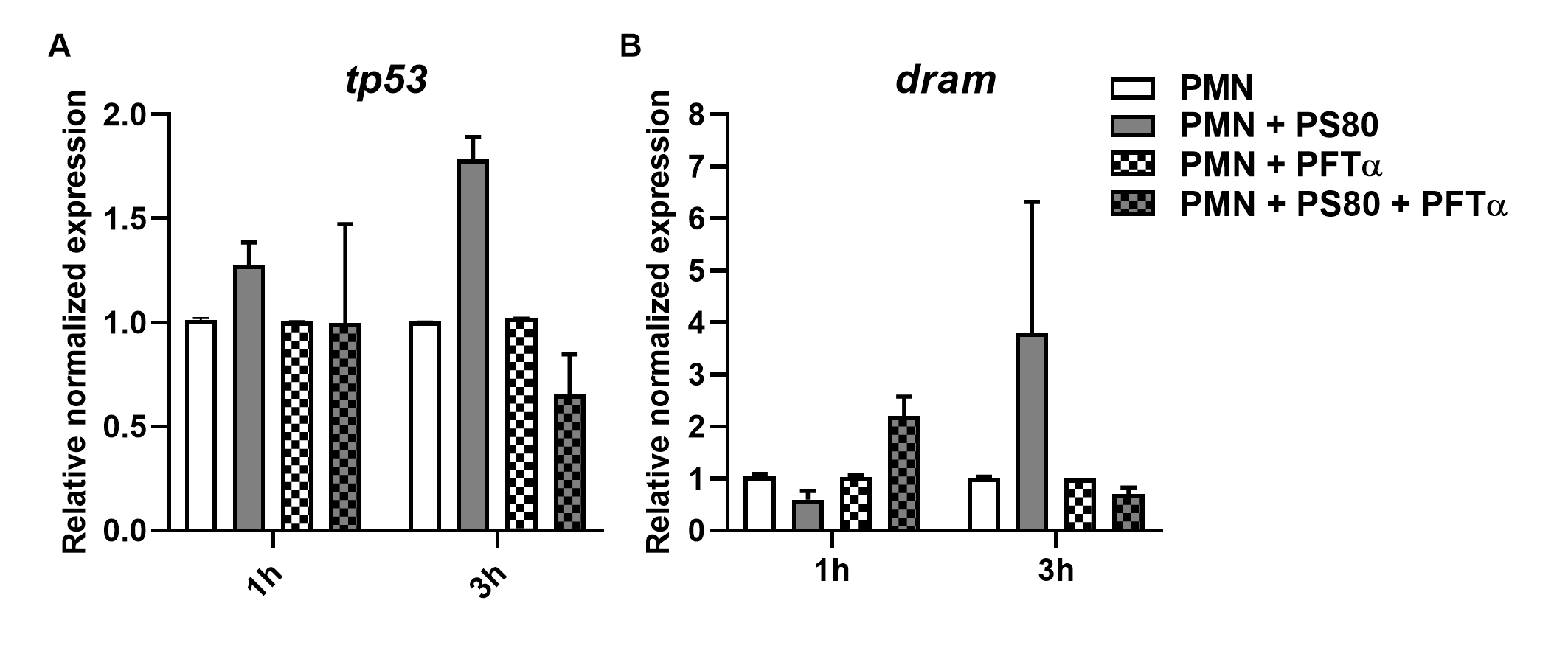

Supplement: Supplementary Figure 4 — tp53 and dram expression after Pifithrin-α treatment during Staphylococcus aureus intracellular survival in neutrophils. Primary human neutrophils were treated with Pifithrin-α (30μM) or were left untreated and were then infected with pre-opsonized S. aureus PS80 (MoI 10) for 1 h. Following infection, PMN were treated with gentamicin (200 µg/ml) for the times indicated. RNA was extracted and gene expression levels of (A) tp53 and (B) dram were assessed using quantitative RT-PCR. Gene expression is plotted relative to gene expression in control PMN after normalization to 18s RNA ± SEM (n = 2 donors). [file Image_4.tif]
